# What is the Effect of Stimulus Complexity on Attention to Repeating and Changing Information in Autism?

**DOI:** 10.1007/s10803-021-04961-6

**Published:** 2021-03-19

**Authors:** Iti Arora, Alessio Bellato, Teodora Gliga, Danielle Ropar, Puja Kochhar, Chris Hollis, Madeleine Groom

**Affiliations:** 1 Division of Psychiatry and Applied Psychology, School of Medicine, University of Nottingham, Institute of Mental Health, Innovation Park, Triumph Road, Nottingham, NG7 2TU United Kingdom; 2 School of Psychology, University of East Anglia, Norwich, NR4 7TJ UK; 3 School of Psychology, University of Nottingham, University Park, Nottingham, NG7 2RD UK; 4 NIHR MindTech Healthcare Technology Co-operative, Institute of Mental Health, University of Nottingham, Nottingham, NG7 2TU UK; 5 NIHR Nottingham Biomedical Research Centre, Institute of Mental Health, Innovation Park, Triumph Road, Nottingham, NG7 2TU UK

**Keywords:** Autism, Eye-tracking, Habituation, Information foraging, Autistic traits

## Abstract

Slower habituation to repeating stimuli characterises Autism, but it is not known whether this is driven by difficulties with information processing or an attentional bias towards sameness. We conducted eye-tracking and presented looming geometrical shapes, clocks with moving arms and smiling faces, as two separate streams of stimuli (one repeating and one changing), to 7–15 years old children and adolescents (n = 103) with Autism, ADHD or co-occurring Autism+ADHD, and neurotypical children (Study-1); and to neurotypical children (n = 64) with varying levels of autistic traits (Study-2). Across both studies, autistic features were associated with longer looks to the repeating stimulus, and shorter looks to the changing stimulus, but only for more complex stimuli, indicating greater difficulty in processing complex or unpredictable information.

**Supplementary Information:**

The online version contains supplementary material available at 10.1007/s10803-021-04961-6.

## Introduction

Autism Spectrum Disorder (hereafter referred to as autism) affects an estimated 1% of the population in the UK (Laurie & Border, 2020) and is characterised by impairments in social communication and interaction and presence of repetitive and restricted behaviours (American Psychiatric Association, 2013). Autistic individuals show atypical attention to the world, for example, in the form of reduced spontaneous attention to social information (Fletcher-Watson et al., 2009; Franchini et al., 2017), an intense focus on specific aspects of the world (American Psychiatric Association, 2013), and a preference for repetition and sameness (Pierce et al., 2011). However, the exact nature of attentional differences, and what processes or impairments underlie them, remains unclear. It has been suggested that early differences in the ability to habituate might contribute to some of the above attentional features (Ramaswami, 2014; McDiarmid et al., 2017).

Habituation refers to a cognitive process by which attention to a repeating stimulus decreases over time (Groves & Thompson, 1970; Schmid et al., 2014). Traditionally, habituation has been studied through preferential-looking paradigms in which look durations are measured to repeated presentations of a stimulus (Csibra et al., 2016). Look durations (i.e. durations of time that the participant orients their eyes to fixate upon a stimulus) in such paradigms measure the balance between a drive to look and a competing drive to look away (Schoner & Thelen, 2006). Widely accepted models of habituation (Groves & Thompson, 1970) suggest that look durations to a repeating stimulus increase until an internal representation has been formed that matches the stimulus (and thus, the stimulus has been ‘learnt’), after which, look durations decrease until they reach an asymptotic level. Look durations in these paradigms have been reliably linked with information processing and learning, such that higher rates of decrease in look durations (or quicker habituation) are associated with better long term outcomes on standardized measures of intelligence (Colombo & Mitchell, 2009); and individual differences in habituation during the first year of life predict later cognitive functioning, including in domains such as language, memory and spatial reasoning (McCall & Carriger, 1993). Given these relationships with other cognitive functions, it is important to understand differences in habituation more fully as these differences may contribute to other cognitive features of autism.

It is also theorized that the drive to look away from an already processed stimulus within such habituation paradigms represents a novelty bias; a pervasive information foraging tendency in all animals that serves an adaptive function of drawing attention away from what is known, towards what is novel, unknown and potentially informative (Schoner & Thelen 2006; Laucht et al., 2006; Cohen et al., 2007). Indeed, from infancy onwards, a balance between exploitation (of the known) and exploration (of the unknown) is essential for optimal adaptation to the environment so that one is alert to pertinent new information but at the same time can focus on a given task (Cohen et al., 2007). If there is a bias towards exploitation or exploration, this could impact optimal foraging and, consequently, learning and adaptive functioning (Gliga et al., 2018).

There is evidence for reduced habituation in autistic individuals for both simple stimuli (e.g., tones and naturalistic sounds (Guiraud et al., 2011; Hudac et al., 2018) and more complex stimuli such as faces (Kleinhans et al., 2016; Webb et al., 2010)). However, it is unclear whether atypical habituation in autism is driven by impaired information processing, leading to slower learning/acquisition of knowledge about the repeating stimulus, or an information foraging style that biases against novelty and change in favour of sameness and predictability. Evidence that habituation deficits in autism are specific to certain stimuli (present for faces but not for houses) (Webb et al., 2010; Kleinhans et al., 2016) implicates slower processing of a repeated stimulus rather than biases against novelty, because complex stimuli, such as dynamic, multimodal and social stimuli, are more difficult to process and would therefore challenge these basic learning processes more extensively. On the other hand, there is evidence of an attentional bias away from novelty, and towards attending to previously explored information at the cost of attending to unknown information (Sasson et al., 2008; Pellicano et al., 2011; Elison et al., 2012). Currently, it remains unknown whether looking longer at a repeating stimulus reflects impaired learning of the stimulus or a preference for repetition. In the habituation literature, it is not possible to disentangle these competing accounts because only a single, repeating stimulus is usually presented and therefore an attentional bias towards repetition over novelty cannot be measured. Whether impaired learning or repetition preference underlies longer looking to a repeating stimulus has important implications for theoretical understanding of autism as well as clinical interventions. Early differences in attention impact the development of socio-cognitive skills that lie at the core of autism (Keehn et al., 2013). If atypicalities in information processing underlie differences in attention, interventions targeting information processing generally could be effective in improving long-term outcomes. If on the other hand, profiles of novelty avoidance/repetition preference underlie differences in social attention, this might reflect differences in reward processing and/or arousal regulation (Frank et al., 2009; Jepma et al., 2012); and interventions that target arousal and reward processing networks might be more appropriate.

To separate out these competing accounts we adapted an eye-tracking paradigm that was first published by Vivanti et al. (2018), in which two competing stimuli are presented simultaneously in the left and right parts of a screen, one of which remains constant while the other one changes. The advantage of this paradigm (instead of traditional paradigms that present only a repeating stimulus) is that one can capture competing drives to look at the repeating versus novel stimuli. In the first few trials, preference for either stimuli is likely to not be evident. However, over trials, habituation should occur to the repeating stimulus and preferential looking towards the changing stimulus should increase. The novelty bias, i.e., increased attention to the changing stimulus, thus becomes more prominent after successful learning or processing of the repeating stimulus (Fantz, 1964). Using this paradigm, Vivanti et al. (2018) reported that autistic pre-schoolers required more trials than neurotypical controls to meet habituation criterion, thus exhibiting slower habituation. Using rates of change in total fixation durations per trial to the repeating and changing stimuli, they also reported that while the autistic children (similarly to neurotypical toddlers) showed reduced looking to the repeating information over successive trials, they also showed reduced looking to the changing stimulus over time, whereas neurotypical toddlers increased looking to the changing stimulus. The authors interpreted this to reflect a reduced bias to attend to novelty in autistic participants, rather than an effect of slower learning. However, one could argue that if autistic children were slower to process the repeating stimulus as evidenced by slower habituation, they would then also have been slower to show preference for the changing stimuli. Therefore, this effect (reduced looking to the changing stimulus) could be driven by slower habituation rather than reduced preference for novelty. Further work is needed therefore to fully characterise profiles of habituation and novelty biases in autism.

**Fig. 1:**
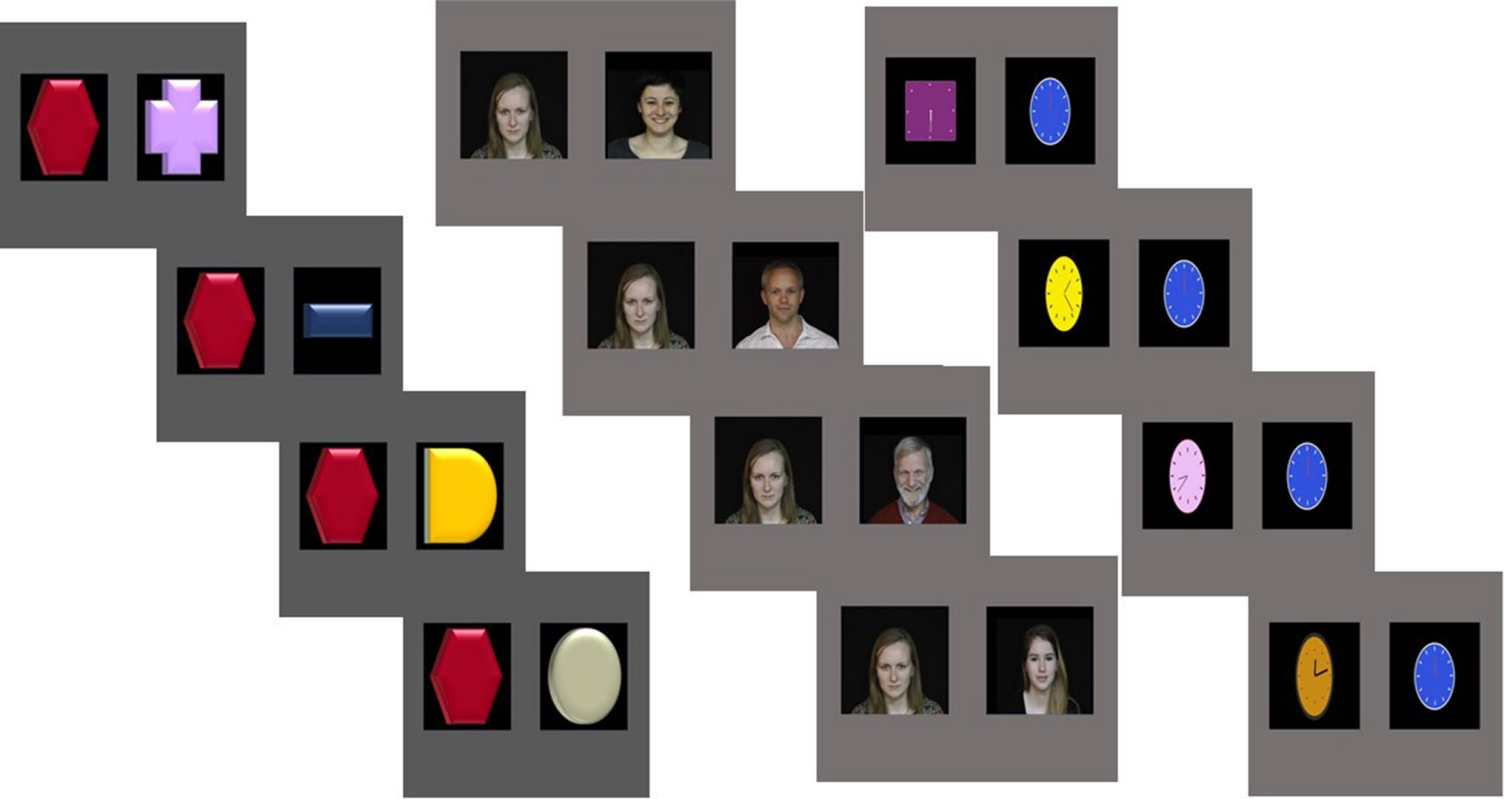
Examples of stimuli used. From left to right, examples of stimuli from non-social simple condition, social condition and non-social complex condition

One way to directly address the role of information processing is by manipulating stimulus complexity. Simpler stimuli elicit quicker habituation than complex stimuli (Schoner & Thelen, 2006). We reasoned that if autistic people tend to spend longer looking at a repeating stimulus because they are slower to habituate, more complex stimuli, which require more processing, should elicit a greater differential between repeating and changing stimuli. Conversely, if the findings are driven by information foraging differences in autistic individuals that bias them against attending to novel or changing information, this will be reflected in a significantly greater proportion of time looking towards the repeating stimulus than the changing stimulus and this effect will occur irrespective of the complexity of the stimulus. To investigate these alternative predictions, we adapted the task used by Vivanti et al. (2018), which comprised one stimulus condition with simple shapes that rotated and zoomed towards the participants. We added two conditions: one consisted of complex stimuli (clocks with moving arms); another used social (smiling faces) stimuli (as shown in Fig. 1). These manipulations allowed us to test whether differences in attention to repeating and changing stimuli were more pronounced for complex than simple stimuli and also allowed us to test whether these effects were more pronounced for social stimuli, given the large literature suggesting greater impairments in the social domain in the autistic population (Dawson et al., 2012; Chita-Tegmark, 2016). We reasoned that if social stimuli are one example of complex stimuli, the faces and clocks stimuli used in our adapted habituation paradigm should yield similar effects to one another, and larger effects than the simple shapes condition. If, however, autistic individuals show a unique difficulty with social stimuli, the effects would be specific to this condition, over and above those for the non-social simple (shapes) and non-social complex (clocks) conditions. Faces and clocks were selected as social and non-social examples of more complex stimuli because they have a higher number of features to process, that hold informative value compared to the geometric shapes.

In addition, we developed a more sensitive measure to capture habituation. Vivanti et al. (2018) used a total fixation duration measure; however, in a two-stimulus habituation paradigm, this measure might also capture other processes apart from information processing, such as revisits to the repeating stimulus to ensure that it has not changed, or even a preference for repetition. We therefore chose to use the longest look duration per trial (comprised of one or more fixations within a stimulus) to each stimulus (repeating and changing). This is more likely to reflect looks made for the purpose of information processing and learning in a given trial (Colombo & Mitchell, 2009). We summarised the pattern of change in look durations over trials by using a slope coefficient, with decreases in look durations reflected in a negative coefficient and increases in a positive coefficient. At the beginning of the task, we expected to observe equally long look durations to both the repeating and changing stimuli. If a person is habituating, then over time, the trial-by-trial longest look durations should decrease for the repeating stimuli and increase for the changing stimuli, since the latter hold novel information. If there is a bias for either the repeating or changing stimulus, this will emerge as an increase in look durations towards that stimulus over time.

In neurotypical individuals, we predicted a rapid decrease in longest look durations to the repeating stimulus over time and an increase in longest look durations to the changing stimulus over time, reflecting rapid habituation and then an information foraging drive towards the novel stimulus. This would be reflected in a negative slope coefficient of look durations to the repeating stimulus and a positive slope coefficient to the changing stimulus. In autism, we predicted that if the tendency to spend longer looking at a repeating stimulus is driven by slower information processing (and therefore slower habituation), there will be a reduction in look durations over time to the repeating stimulus and an increase to the changing stimulus, but the slopes will be flatter than in neurotypical individuals, reflecting slower change over time. This effect will be more pronounced in the conditions with higher stimulus complexity due to the greater difficulty processing these stimuli. Conversely, if driven by a bias against novelty towards sameness, the effect will not vary by stimulus complexity and will manifest in a significant positive slope to the repeating stimulus and a flat or negative slope to the changing stimulus, i.e. a reversal of the neurotypical effect. We also explored whether these atypical features of autism are specific to social stimuli or whether they also occur when presented with non-social stimuli that have a similar level of featural complexity.

We used this task with two populations. In Study 1, we compared children with and without clinically diagnosed autism and we also compared autism with another neurodevelopmental disorder, attention deficit hyperactivity disorder (ADHD). In Study 2 we recruited a general population sample of children with varying levels of autistic traits.

## Study 1

The aim of the first study was to determine whether differences in attention to repeating vs changing stimuli reflect slower processing of a repeated stimulus or atypical biases away from novelty in autistic children, by manipulating stimulus complexity. Therefore, in this study, we included children with a clinical diagnosis of Autism Spectrum Disorder and neurotypical children. In addition, we included a group of children with ADHD and a group of children with co-occurring Autism and ADHD.

ADHD is highly co-occurrent with autism (with co-occurrence rates between 37 and 85%, Leitner 2014) but this is often not addressed in research. There is inconsistent evidence for atypical habituation in ADHD; with preliminary evidence for quicker habituation to rewards in those with ADHD (McDiarmid et al., 2017). ADHD is also tentatively associated with biases towards novelty-seeking and exploration (Gliga et al., 2018) and could therefore be linked with information foraging biases opposite to the ones associated with autism. Given the high comorbidity between these conditions, investigating how these potentially opposing biases are manifest in those with comorbidity might illuminate shared mechanisms between autism and ADHD. Therefore, the aim of our first study was to determine how attention to repeating vs changing information is influenced by stimulus complexity and whether any unique attentional patterns are evident within different clinical groups with a diagnosis of autism, ADHD, or both. In many experimental studies on autism, despite the high levels of co-occurrence between autism and ADHD, co-existing ADHD is either ignored (not measured) or autistic participants are excluded from the studies if they meet criteria for ADHD. This reduces the generalizability of results from those studies, as their samples are not representative of the general autistic population. Instead, careful characterization of ADHD symptoms in autistic participants provides an opportunity to test how presence of ADHD impacts profiles of attention and information processing in autism and in doing so, we are also able to include a more representative sample of autistic children and young people in the study.

We predicted a profile of relatively greater attention to the repeating stimulus over the changing stimulus in children and adolescents with autism, as outlined in the general introduction above. For children with ADHD, our hypotheses were more tentative, given that such tasks have not been used with this population before. We expected them to show a bias towards novelty, to the extent that they will look more often at the changing stimulus (Sethi et al., 2018). We also expected, given profiles of hyperactivity and inattention (American Psychiatric Association, 2013), that they might be slower to reduce their attention to repeating information due to inefficient processing and therefore, flatter slopes of change in attention towards both stimuli. Again, given lack of research in the area, we anticipated different possible effects for children with co-occurring autism and ADHD. Given evidence of opposing information foraging biases in autistic and ADHD populations (towards novelty in ADHD and against novelty or towards sameness in autism), we anticipated that comorbid children might show neither, with the two opposing risks combating each other. Alternatively, the group with co-occurring autism and ADHD might be more similar to the autistic children, or to the ADHD children, reflecting that on these measures they share the profile of one of these populations. Finally, the comorbid group might be a separate nosologic entity and thus might show a completely distinct profile (Rommelse et al., 2011) from the other children. We tested these predictions in a factorial design where ADHD and ASD were modelled as two between-subjects’ factors.

## Methods

### Sample

The present work is based on data collected for the SAAND study (Studying Attention and Arousal in children and adolescents with Neurodevelopmental Disorders). 103 participants aged 7–15 years took part, including 30 neurotypical participants, 18 with Autism, 23 with ADHD and 32 with both Autism and ADHD (‘Autism+ADHD’). Participant demographic characteristics are presented in Table 1.

**Table 1:** Sample characteristics for study 1

	Neurotypical (n = 30)	Autism (n = 18)	ADHD (n = 23)	Autism + ADHD (n = 32)	Group Comparisons (p value)
Demographics					
Age	129.63 (29.29)	130.89 (25.05)	127.87 (27.14)	130.06 (18.36)	Ns (p^w^>.1)
Gender M:F	17:13	11:7	15:8	24:8	Ns (p^w^>.1)
WASI full-scale IQ	116.2 (13.34)	104.61 (15.64)	108.61 (11.67)	102.06 (19.29)	p^w^ = 0.006^a^
SCQ					
Total	3.79 (3.71)	19.11 (5.98)	15.17 (6.96)	21.16 (6.23)	p^w^ < 0.001^b,c^
SCQ Social	1.25 (1.5)	7.56 (3.34)	4.91 (3.26)	7.68 (3.47)	p^w^ < 0.001^b,c^
SCQ Comm	1.82 (1.49)	5.61 (2.3)	4.61 (1.99)	6.39 (2.33)	p^w^ < 0.001^b,c^
SCQ RRB	0.5 (1.1)	4.56 (2.2)	4.04 (2.51)	5.42 (2.76)	p^w^ < 0.001^b^
CPRS					
Global Index	51.82 (13.45)	79.44 (12.59)	87.87 (4.25)	87.13 (5.32)	p^w^ < 0.001^b^
Inattention	50.57 (9.75)	77 (12.48)	86.78 (6.64)	85.09 (6.41)	p^w^ < 0.001^b,d^
Hyperactivity	52.32 (12.93)	76.44 (13.68)	87.83 (3.9)	87.38 (5.56)	p^w^ < 0.001^b,e^

Data shown for all measures except Gender are mean with standard deviation in parentheses. Data for gender are n male:female. WASI: Wechsler Abbreviated Scale of Intelligence; CPRS: Conners Parent Rating Scale (values shown are mean T-scores); SCQ: Social Communication Questionnaire

p values in the table refer to the significance value of the main ANOVA, comparing the 4 groups on respective demographic characteristics; multiple comparisons for these variables are Bonferroni-corrected. p^w^ refers to the p value of Welch’s F test carried out where homogeneity of variances assumption was violated; for these variables, post-hoc comparisons are corrected using Games-Howell method

^a^NT>Autism+ADHD

^b^NT < Autism, ADHD, Autism + ADHD

^c^ADHD < Autism+ADHD

^d^Autism < ADHD

^e^Autism < ADHD, Autism + ADHD

Participants completed a battery of EEG and eye-tracking tasks, including the task presented here. Study procedures were approved by the UK National Research Ethics Committee (REC reference 17/EM/0193 and the Health Research Authority (HRA; IRAS research project ID 220158). Clinical participants were recruited through local support groups or were referred to the study by paediatricians, child and adolescent psychiatrists or mental health nurses in local Child and Adolescent Mental Health Services (CAMHS) or the special needs departments of local schools. Neurotypical participants were recruited from local schools and from a database of volunteers held by the School of Psychology, University of Nottingham, UK. Participants in the clinical groups either already had a clinical diagnosis or were referred to the study by clinicians because of suspected ADHD or autism. Consensus research diagnoses were made in consultation with two experienced child and adolescent psychiatrists (PK and CH). The measures used to inform research diagnoses were: Development and Well-Being Assessment (DAWBA) (Goodman et al., 2000), Social Communication Questionnaire (SCQ) (Rutter et al., 2003), Conners’ Rating Scales (CRS-3) (Conners, 2008), the Autism Diagnostic Observation schedule, 2nd edition (ADOS-2) (Lord et al., 2015) (completed by IA and PK who have research accreditation for the tool) and the Wechsler Abbreviated Scales of Intelligence (WASI-II) (Wechsler, 2011) to obtain a measure of verbal and non-verbal cognitive functioning for all participants. Parent and teacher data were available for the participants on the SCQ and CRS-3. Due to missing data on the teacher measure, in this study we report the parent CRS scores. In this study, we used parent-reported SCQ (Total score and social communication, social interaction and restricted and repetitive behaviours subscale scores) and CRS (Hyperactivity-Impulsivity and Inattention subscales) scores as indices of symptom severity of Autism and ADHD respectively. Further information about inclusion/exclusion criteria as well as allocation of participants into clinical groups is available in Supplementary Materials.

### Eye-Tracking Task

We adapted the novelty versus repetition task from Vivanti et al. (2018). In this task, two streams of dynamic stimuli are presented adjacent to one another, one each in the left and right sides of the screen, on a computer screen. In one stream, a repeating stimulus is presented and in the other, a changing stimulus is presented. In the original task (Vivanti et al., 2018), the stimuli were dynamic shapes, rotating and looming towards the viewer. Stimulus duration was three seconds. We adapted these original stimuli but retained the timing and display parameters of the original study.

In addition, we added two conditions to enable us to measure the effects of social-ness and complexity of stimuli (see Fig. 1). We added a social condition in which the stimuli consisted of movies of faces breaking into smiles taken from the UvA-NEMO Smile Database (Dibeklioğlu et al., 2015). The videos are shot under controlled illumination conditions and are in RGB colour. We cropped the videos to size them similarly to the stimuli from other conditions.

We also created a non-social condition in which we used animations of clocks with moving arms as stimuli. Clocks were sized similarly to the faces in the social condition. Clocks were of different colours (similar to non-social simple condition), and the arms moved from different starting points to different endpoints. The clocks were designed to be more complex than the shapes since there was more information within them to process. Clocks have multiple features that have informative value and the movement of internal features changes the meaning to be drawn from the stimulus, similar to facial features. Importantly, the faces and clocks differ primarily in their social status but are approximately equivalent in global and featural complexity (see Fig. 1).

In keeping with the original study (Vivanti et al., 2018), we chose to use dynamic stimuli for our other two conditions. This was primarily because, for the age range of our participants, static stimuli would have been too simple and possibly unengaging. Furthermore, dynamic stimuli are more naturalistic and therefore have greater ecological validity. In Vivanti et al. (2018) study, nine trials were presented. We added two trials (to each condition) to ensure that there were sufficient trials to capture changes in looking patterns given the older age of our participants. Therefore, in Study 1, each condition comprised of eleven trials (3 s per trial), leading to three conditions that lasted 33 seconds each, and an entire task that lasted around 2–3 min in total, including calibration and drift correction between conditions. For each stimulus type, there were twelve stimuli created, one of which was used as the repeating stimulus while the rest were used as changing stimuli, so that within the changing stimuli, no stimulus was presented more than once. Order of presentation of conditions and stimuli within conditions were both randomized. Further, we counterbalanced the visual hemifield in which the repeating stimulus was presented in each condition and between the two versions.

Further information about task design is available in Supplementary Materials.

### Procedure

The task was delivered on Eyelink 1000 Plus after a 9-point gaze calibration was completed. Eye movements from both eyes were recorded without a chin-rest and children were seated approximately 60 cm from the screen. Eye movements were recorded at 500 Hz through a 25 mm lens, with an estimated accuracy of 0.25° to 0.5°. The task was presented on a 21.5’’ LCD screen with a refresh rate of 60 Hz, placed immediately behind the eye-tracker.

This task lasted approximately 2 minutes, including calibration. It was a part of a 15-minute eye-tracking battery and was presented mid-way through another eye-tracking task. Participants were asked to pay attention to what was happening on the screen but were given no other instructions.

### Analysis Plan

We extracted two measures from the task. The first, number of fixations to the screen, was a measure of task engagement, compared between groups to ensure that analysis of other measures was not influenced by any between-subject differences in task engagement. The second measure of interest was the rate of change in look durations to the repeating and changing stimulus over time. Interest areas were drawn around stimuli to capture any fixations falling within the area of the stimuli. A ‘look duration’ was defined as cumulative duration of consecutive fixations in the same interest area in a trial without shifting to another interest area. Therefore, for each trial, the longest look to the repeating and changing stimulus was extracted. We then computed the coefficients of the linear slope of the rate of change in these look durations to the repeating and changing stimulus in each condition (Non-Social Simple, Non-Social Complex, Social) separately. We expected a negative slope to the repeating stimulus across conditions, representing reduced looking to repeating information over time, and a positive slope to the changing stimulus, driven by longer looking to the changing information over time representing a novelty bias.

In the main analyses, Autism and ADHD were modelled as two between-subject factors with two levels each, ‘Present’ and ‘Absent’. This allowed us to measure the effects of either condition separately through main effects of either factor. Modelling the factors in this way gave more power to the comparisons when comparing all participants with Autism/ADHD with those without. Effects specific to one of the four groups would emerge in this analysis through an interaction effect between the between-subject factors, and this would allow us to investigate whether a profile of attention was specific to the autism only group as compared to the rest.

To analyse the engagement variable (number of fixations), we used repeated measures analyses of variance (ANOVA) with one within-subject factor: Condition with three levels (Non-Social Simple, Non-Social Complex, Social). In our analysis of this variable we focussed on checking individual differences in task engagement. We therefore only report main effects of Autism or ADHD or interactions between these and the within-subjects Condition factor. For our main analysis on the Rate of change in Look durations, we included a second within-subjects factor Stimulus with two levels (Repeating, Changing).

For each dependent variable, we assessed common assumptions before testing hypotheses. Mahalanobis distances were used to identify multivariate outliers but none were identified. Based on evidence that repeated measures ANOVAs are robust to assumptions of normality we carried out ANOVA with normal and non-normal dependent variables (Field 2013). Mauchly’s tests of sphericity was evaluated and where violated, we report Greenhouse-Geisser adjusted degrees of freedom. Interactions and main effects were followed up with appropriate analysis to characterise the simple effects.

Given differences between clinical groups on IQ, we used partial correlations to evaluate whether differences in IQ were associated with any effects of interest.

## Results

Overall, the pattern of group differences reflected the group allocations, showing greater CRS scores in the ADHD and Autism+ADHD groups and greater SCQ scores in the Autism and Autism+ADHD groups. The clinical groups had lower IQ than the neurotypical group; however, this difference was statistically significant only between NT and Autism + ADHD group (see Table 1).

### Number of Fixations (Control Variable Measuring Task Engagement)

First, we analysed participants’ number of fixations to the screen to ensure that all participants were attentive to the task at all levels of Condition. The between-subjects factor of Autism interacted significantly with Condition: F (2, 198) = 3.03, p = 0.05, ƞ^2^_p_ = 0.03. However, follow up pairwise comparisons comparing groups (Autism-Present, Autism-Absent) within each condition yielded no significant differences (all p > 0.1) (descriptive statistics provided in Supplementary Materials). Main effects of Autism and ADHD were not significant: Autism: F (1, 99) = 0.008, p = 0.93, ƞ^2^_p_ = 0.00; ADHD: F (1,99) = 0.009, p = 0.92, ƞ^2^_p_ = 0.00.

### Rate of Change in Look Durations

We predicted that all participants would show reduced look durations over time to the repeating stimulus (indexed by a negative slope) and increased look durations over time to the changing stimulus (indexed by a positive slope). There was a main effect of Stimulus (F (1, 99) = 52.78, p = 0.000, ƞ^2^_p_ = 0.35). As predicted, this was driven by a significantly more positive slope for the changing stimulus (Mean ± SE = 40.04 ± 4.84) as compared to the repeating stimulus (Mean ± SE = −10.84 ± 3.68). There was also a main effect of Autism (F (1, 99) = 4.74, p = 0.032, ƞ^2^_p_ = 0.046). This was driven by those without Autism (neurotypical and ADHD-only: Mean ± SE = 20.03 ± 3.42) showing steeper slopes than those with Autism (Autism-only and Autism+ADHD: Mean ± SE = 9.17 ± 3.63).

**Fig. 2:**
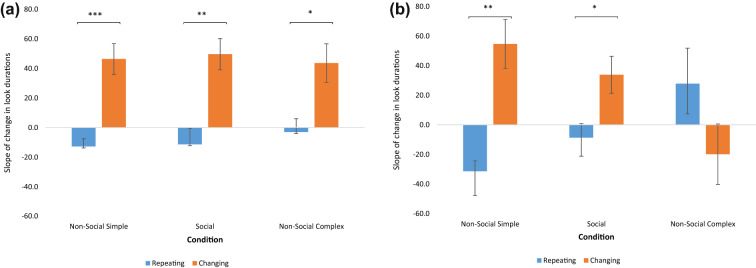
**a** The main effect of stimulus in neurotypical participants. Bars show the mean (±1 standard error) coefficient of the slope for the rate of change in look durations over trials (plotted on the y-axis). These data are split by stimulus type and condition. Asterisks denote statistical significance: *p < 0.05, **p < 0.01, ***p < 0.001. The interaction between Condition. *Stimulus is non-significant but shown here for the purpose of visualization of differences from the Autism-only group shown in Fig. 2b. **b** Condition*Stimulus interaction in the autism-only group. Bars show the mean (±1 standard error) coefficient of the slope for the rate of change in look durations over trials (plotted on the y-axis). These data are split by stimulus type and condition. Asterisks denote statistical significance: *p < 0.05, **p < 0.01, ***p < 0.001

There was an interaction between Condition and Stimulus (F (1.87, 185.25) = 8.74, p < 0.001, ƞ^2^_p_ = 0.08) driven by a significant main effect of Stimulus for the Non-Social Simple (Mean difference Repeating vs Changing = −82.38 ± 11.16, p < 0.001) and Social (Mean difference = −53.74 ± 9.93, p < 0.001) conditions, which was non-significant in the Non-Social Complex condition (Mean difference = −16.51 ± 13.18, p = 0.213). This two-way interaction was moderated by a 4-way interaction between Condition*Stimulus*Autism*ADHD: F (1.87, 185.25) = 3.82, p = 0.026, ƞ^2^_p_ = 0.037. We broke this interaction down by running two repeated-measures ANOVAs, separately within each level of Autism and within each level of ADHD. At each level of Autism (Absent, Present), the three-way Condition*Stimulus*ADHD interaction was not significant: Autism-Absent: F (2, 102) = 1.49, p = 0.23, ƞ^2^_p_ = 0.028; Autism-Present: F (1.78, 85.55) = 2.39, p = 0.103, ƞ^2^_p_ = 0.047. The equivalent analysis at each level of the ADHD factor showed that the three-way Condition*Stimulus*Autism interaction was not significant at ‘ADHD-Present’: F (2, 106) = 1.18, p = 0.308, ƞ^2^_p_ = 0.022; but, in the groups without ADHD (that is in the neurotypical (NT) and Autism-only groups), there was a three-way interaction of Condition*Stimulus*Autism (F (2, 92) = 4.375, p = 0.015, ƞ^2^_p_ = 0.087). Follow-up comparisons were conducted to test the Condition*Stimulus interaction in each of these groups (NT, Autism-only). These analyses showed a significant main effect of Stimulus in Neurotypical children (p < 0.0001, ƞ^2^_p_ = 0.447), with shorter looks to repeating stimuli (Mean ± SE = −9.03 ± 5.5) and longer looks to changing stimuli (Mean ± SE = 46.49 ± 7.74) over time across conditions (see Fig. 2a); the Condition*Stimulus interaction was not statistically significant in this group (F (2, 58) = 0.29, p = 0.75). On the other hand, the Condition*Stimulus interaction was significant in the Autism-only group (F (2, 34) = 5.50, p = 0.009, ƞ^2^_p_ = 0.24) with shorter look durations over time to the repeating stimulus and longer look durations over time to the changing stimulus in the Non-Social Simple (repeating vs changing Mean ± SE: −31.39 ± 7.03 vs. 54.64 ± 16.48) and Social conditions (repeating vs changing Mean ± SE: −8.68 ± 9.53 vs. 33.77 ± 12.52) but a numerical difference in the opposite direction in the Non-Social Complex condition which did not reach statistical significance (repeating vs changing Mean ± SE: 27.79 ± 23.96 vs −19.88 ± 20.41) (as shown in Fig. 2b).

### Correlations with SCQ

**Fig. 3:**
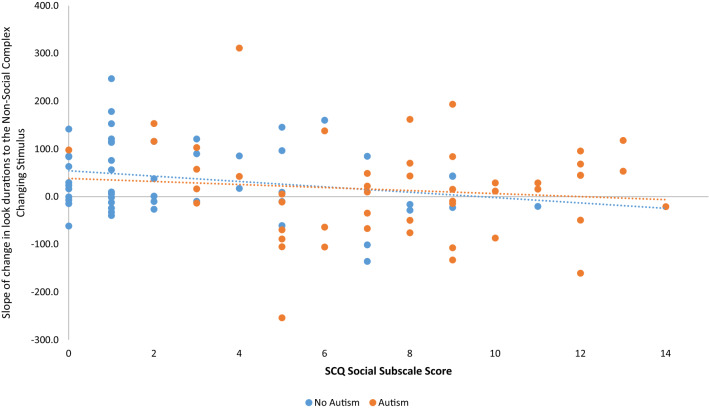
Relationship between SCQ-Social scores and rate of change measure in non-social complex condition*.* Scatterplot of scores on Social Communication Questionnaire (SCQ) Reciprocal Social Interaction Subscale (plotted on the x-axis) with the coefficient of the slope for the rate of change in look durations over trials to the Non-Social Complex Changing Stimulus (plotted on the y-axis) for participants with and without Autism (represented by orange and blue dots respectively. Dotted orange and blue lines represents the trend lines for the participants with and without Autism respectively

Bootstrapped bivariate correlations were computed between number of fixations to repeating and background stimuli (across conditions) and rate of change of attention to the repeating and changing stimuli in the non-social complex condition) and the SCQ subscales of social, communication and RRB symptoms. A greater reduction in look durations to the changing stimulus over time in the Non-Social Complex condition was associated with higher SCQ Social symptoms (r = −0.198, p = 0.05, [−0.365, −0.032]) (See Fig. 3), suggesting that those with higher symptom severity on this scale showed a bias against attending to the changing stimulus over time, in this condition. To evaluate the role of IQ, we computed partial correlations between SCQ Social symptoms and Rate of change of attention to the changing stimulus in the Non-Social Complex Condition, whilst controlling IQ. The correlation became nonsignificant (r = −0.161, p = 0.112, [−0.326, −0.007]).

Given the finding of flatter slopes for the rate of change in look durations overall in autistic individuals as compared to non-autistic individuals in our sample, we also ran a correlation between IQ and the average rate of change of look durations over time with data collapsed across conditions and stimuli. The correlation was not statistically significant (r = −0.111, p = 0.264, [−0.282, 0.079]).

## Summary and Discussion of Study 1

In this study, we set out to identify whether differences in attention to repeating versus changing information in autism are present across stimulus contexts, suggesting a bias away from novelty towards repetition and predictability; or if they are dependent upon stimulus complexity, indicating slower information processing which is exacerbated when stimuli are complex. Further, we investigated whether this attention profile was specific to children with autism when compared with a group of children with ADHD. Finally, we also included a group of children with co-occurring autism and ADHD to investigate what profile of information foraging biases they show.

Analysis of the rate of change in look durations to the repeating versus changing stimuli revealed that autistic participants (with or without ADHD) showed flatter slopes of change in look durations to repeating and changing stimuli across conditions of stimulus complexity, suggesting that they were slower to shift attention, possibly due to slower information processing. Further, autistic children (without co-occurring ADHD) showed a neurotypical profile of reduced attention over time to the repeating stimulus and increased attention over time to the novel stimulus in the Non-Social Simple (shapes) and Social conditions. However, they did not show this effect in the Non-Social Complex (clocks) condition, in which they showed prolonged attention to the repeating over the changing stimulus. This is a reversal of the neurotypical effect and indicates that autistic children are not just defined by reduced habituation to a repeating stimulus but, when presented with visually complex stimuli, they show a bias towards repetition and away from novelty. This effect is more complex than we predicted as it suggests both slower information processing, reflected in flatter slopes to the repeating and changing stimuli (compared with neurotypical participants) with a preservation of the changing>repeating pattern to Social and Non-Social Simple stimuli, and a bias for repetition over novelty (reflected in a reversal of the changing>repeating effect) to Non-Social Complex stimuli. This is an important effect, which suggests that attentional biases in favour of exploring known over unknown information (Sasson et al. 2008; Pellicano et al. 2011; Elison et al. 2012) might partly be driven by a response to stimulus complexity such that greater complexity elicits this bias towards sameness and predictability, away from novelty (Kawa and Pisula 2010; Hanley et al., 2013).

Interestingly, although this effect of a bias towards repetition did not occur in the Social condition, the effect in the Non-Social Complex condition was associated with social impairments in our sample, such that those with more parent-reported social interaction difficulties showed an atypical bias away from the changing stimulus in the Non-Social Complex condition. It is interesting that the autistic sample showed a neurotypical profile in the Social condition, albeit with flatter slopes for look durations than the NT group. One possibility is that the social stimuli used here were not complex enough; further work is needed to determine whether more socially complex stimuli (for example multimodal stimuli combining faces with speech) would also elicit the effect found here in the Non-Social Complex clocks condition.

ADHD was not related to any predicted effects. Further, while autistic participants (with or without ADHD) showed flatter slopes of rate of change in attention to both stimuli overall, only those with autism without ADHD showed an additional bias against novelty when stimuli were particularly complex. This suggests that the co-occurring presence of ADHD benefited those with autism, protecting them from biases against novelty in the Non-Social Simple and Social conditions, possibly through a compensatory effect of an opposing bias towards novelty, as suggested by Gliga et al. (2018), who reported that infants at elevated likelihood of both autism and ADHD did not show exploitative biases. However, in our study, given that ADHD was not a main effect in these analyses, we cannot call this an additive effect because we did not find evidence of opposing biases being nulled in the comorbid group.

To summarize, Study 1 found that autistic participants (with and without ADHD) exhibited a slower rate of change in look durations over time as evidenced by flatter slopes, possibly due to slower processing of information. Autistic children (without ADHD) showed a profile of prolonged attention to repetition and reduced attention to the changing stimulus over time, but only in the Non-Social Complex condition. Biases against exploration of new information in complex conditions were associated with higher social impairments in our sample, across autistic and non-autistic participants.

## Study 2

The aim of the second study was to determine whether the effect found in Study 1 (wherein autistic participants’ attention to changing information is reduced only in contexts of higher stimulus complexity) extends into the general population in individuals with high autistic traits. The behavioural profile associated with autism has been found to be present sub-clinically in those at increased familial risk of autism, termed the Broad Autism Phenotype (BAP), (Piven, 2001; Robinson et al., 2011). Further, the autistic traits that comprise the BAP, such as reduced social skills and impaired social cognitive abilities, as well as restrictive and repetitive behaviours, have been found to extend into the general population, suggesting that they lie on a continuum between individuals meeting diagnostic criteria and those in the general population (Constantino & Todd, 2003; Ronald et al., 2006; Ingersoll, 2010; Sasson et al., 2013). Therefore, when teasing apart mechanisms underlying specific features, studying individuals on different sides of the diagnostic boundary may prove fruitful in enhancing our understanding of the autistic spectrum.

We hypothesised that if higher autistic traits are associated with similar risks to information processing, children in our sample with higher autistic traits would orient their attention more towards the repeating stimulus stream over trials, and show reduced attention to the novel stimulus stream; but that this will be specific to conditions where the stimuli are more complex.

## Methods

### Participants

Sixty-four children between the ages of 4–12 years took part in this study (see Table 2 for demographic and behavioural characteristics). Participants were recruited during a local science engagement event (Summer Scientist Week, SSW) organised by the University of Nottingham in 2017 and 2018. Three children were reported to have a pre-existing diagnosis of autism, and one had a pre-existing diagnosis of ADHD. These children were not excluded from analysis as it was considered advantageous to include children on the extreme end of the autism continuum. One child used hearing aids but was not an outlier on any measure so they were included in the analyses.

**Table 2:** Demographic characteristics of the sample in study 2

Demographic	Sample
Sample size	64
Mean age (in months) (SD)	101.797 (23.997)
Gender (M:F)	34 M: 30 F
Mean BPVS (standard score) (SD)	105.16 (11.785)
Mean AQ (SD) (range)	58.33 (18.12) (25–110)

Data shown for all measures except Gender are mean with standard deviation in parentheses. Data for gender are n male:female. *BPVS* british picture vocabulary scale, 3rd Edition; *AQ* autism spectrum quotient- child’s version

### Measures

The British Picture Vocabulary Scale (BPVS3) (Dunn & Dunn, 2009): age-adjusted standard scores (with a mean of 100 and standard deviation of 15) were used as a proxy for mental age. Autistic traits were measured using the Autism Spectrum Quotient- Child’s Version (AQ-Child) (Auyeung et al., 2008), a parent-report questionnaire with high internal consistency (overall alpha = 0.97) and good test-retest reliability (r = 0.85). The AQ-Child has a range of scores from 0-150, with a cut-off score of 76 showing high sensitivity and specificity for Autism.

### Procedure

Ethical approval for the study was granted by the School of Psychology Ethics Committee, University of Nottingham. The eye-tracking task presented to participants was identical to the task described in Study 1 except that, due to time constraints within the SSW experimental set-up, and because the participant sample was recruited from a younger age range, nine trials were presented per condition (similar to the original study by Vivanti et al. (2018)). In the analysis reported here, 13 participants’ data is from 2017, while 51 participants were tested in 2018. Participants received tokens upon completion of the experiment which they could use to spend on games and activities at the event. The equipment used and eye-tracking procedure was the same as that described in Study 1.

### Analysis Plan

We extracted the same two measures as Study 1: Engagement (measured by number of fixations to the screen in different conditions) and the rate of change of cumulative look durations to the repeating and changing stimuli over time in each Condition. The within-subject factors (Stimulus, Condition) were the same as in Study 1.

Here we report the results from our main model testing our hypotheses with AQ score included as a linear predictor. Mahalanobis distances were used to identify multivariate outliers but none were identified. To account for potential effects of factors such as age and mental ability, we ran separate correlations with age and BPVS to assess whether these were related to scores on the AQ-Child and/or task effects of interest.

## Results

### Engagement

First, we analysed participants’ number of fixations to the screen at different levels of Condition (Non-Social Simple, Non-Social Complex, Social) to ensure participants were attentive throughout. AQ did not interact with Condition: Greenhouse-Geisser F (1.77, 109.55) = 0.73, p = 0.47, ƞ^2^_p_ = 0.01. There was also no main effect of AQ scores: F (1, 62) = 0.213, p = 0.65, ƞ^2^_p_ = 0.00.

### Rate of Change in Look Durations

**Figure 4:**
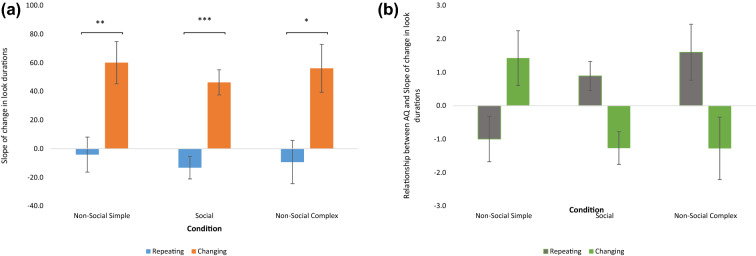
**a** Interaction between Condition and Stimulus on rate of change in look durations. Bars show the mean (±1 standard error) coefficient of the slope for the rate of change in look durations over trials (plotted on the y-axis). These data are split by stimulus type and condition. Asterisks denote statistical significance: *p < 0.05, **p < 0.01, ***p < 0.001. **b** Interaction between Condition, Stimulus and AQ on rate of change in look durations. Bars show the mean (±1 standard error) coefficient of the linear relationship between scores on the Autism Spectrum Quotient- Child Version (AQ-Child) and the rate of change in look durations over trials (plotted on the y-axis). These data are split by stimulus type and condition

There was a main effect of Stimulus (F (1, 62) = 8.16, p = 0.006, ƞ^2^_p_ = 0.116); with the slope to the repeating stimuli being more negative (Mean ± SE = −0.89 ± 6.59) than the slope to the changing stimuli (Mean ± SE = 54.13 ± 7.7). This was modulated by a Condition*Stimulus interaction (Greenhouse-Geisser F (1.8, 111.675) = 4.504, p = 0.013, ƞ^2^_p_ = 0.068). The main effect of Stimulus was present within each condition (See Fig. 4a): Simple (Mean difference (Repeating vs Changing) = −64.13 ± 22.73, p = 0.006); Complex (Mean difference = −65.46 ± 27.99, p < 0.023); Social (Mean difference = −59.56 ± 13.74, p < 0.001). This interaction was further moderated by a 3-way interaction with AQ (F (1.8, 111.675) = 4.96, p = 0.011, ƞ^2^_p_ = 0.074). As can be seen below in Fig. 4b, in both the Non-Social Complex and Social conditions, the main effect of Stimulus reversed, such that in the Non-Social Complex and Social conditions, those with higher AQ scores (i.e., higher levels of autistic traits) showed longer look durations to the repeating stimuli over time and reduced look durations to the changing stimuli over time. Since we included three participants who met criteria for autism and one participant with ADHD in this sample, we also ran this model without those participants to ensure that the results are not an artefact of including clinical participants. Excluding these participants did not change the significance level of any analyses. The results from this analysis are provided in Supplementary Materials.

### Correlations between AQ and Slope of Attention to Repeating and Changing Information

We ran correlations between AQ scores and the slopes of attention to repeating and changing information in the Non-Social Complex and Social conditions. AQ scores correlated positively with the slope of change in longest look durations to the repeating stimulus in the Social condition (r = 0.257, p = 0.044, [.001, 0.502]) and negatively related to the slope to the changing stimulus in the Social condition (r = −0.295, p = 0.02, [−0.48, −0.07]). Thus, higher autistic traits were related to prolonged attention to the repeating stimulus and reduced attention to the changing stimulus in the Social condition.

We then assessed whether any demographic characteristics were related to AQ. Neither BPVS scores nor Age correlated significantly with AQ or with the rate of change in look durations to repeating or changing stimuli in either the Non-Social Complex or Social conditions (all p > 0.1, full correlation values provided in Supplementary Materials).

## Summary and Discussion of Study 2

We aimed to identify whether biases found in our clinical sample of autistic children against attending to changing information when stimuli were more complex are related to autistic traits in a general population sample. Indeed, this is what we found. In the Non-Social Simple (shapes) condition, traits of AQ did not impact information foraging, all children showed the expected profile of reducing attention over time to the repeating stimulus and increasing attention over time to the changing stimulus. However, in the Social (faces) and Non-Social Complex (clocks) conditions, higher traits of AQ were related to reduced look durations to changing stimuli over time and increased look durations to repeating stimuli over time. The presence of this effect for both Social and Non-Social Complex stimuli suggests that, in this study, the two types of stimuli elicit equivalent effects on attention, suggesting that an atypical attentional style to social stimuli may at least partly be explained by the complexity of those stimuli. Our findings are in line with other studies that have investigated social abilities and attention in association with traits of autism (Ingersoll 2010; Sasson et al. 2013) which have also found that higher sub-clinical traits are associated with similar profiles of social abilities as those seen in clinical diagnosis of autism.

## General Discussion

In the present study, we aimed to disentangle whether differences in habituation or biases against novelty drive differences in attention to repeating vs changing information in autistic individuals. We investigated these questions by manipulating stimulus complexity and extracting a measure of information processing and learning, indexed through the longest look duration to each stimulus per trial, to assess how this changed over time to the repeating and changing stimuli. We found that across two independent samples of children, traits and clinical symptoms of autism were related with prolonged attention to repetition and reduced attention to novelty, but only in contexts of higher stimulus complexity (in Non-Social Complex condition in Study 1, and in both Social and Non-Social Complex conditions in Study 2). This suggests that there might be two processes at play: differences in habituation due to difficulties processing more complex stimuli and a bias against novelty in favour of repetition which is elicited by complex stimuli (at least in this paradigm) in individuals with clinical symptoms or higher traits of autism. Our findings are partly in line with Vivanti et al. (2018) report of slower habituation and attentional biases against novelty; however, our findings extend this work by showing that these attention profiles seem to be partly driven by slower learning or processing of stimuli.

Our findings suggest that differences in habituation to repeating stimuli emerge when stimuli are more complex. Importantly, we also found this effect to be specific to children with autism without comorbid ADHD. These are important factors that have previously not been considered in the literature. Studies on habituation mechanisms in autism have yielded heterogeneous findings, with some studies reporting differences in habituation to be only present when using social stimuli (such as faces) but not when using non-social stimuli (Webb et al. 2010; Kleinhans et al. 2016), and interpreting those effects to be related to difficulties in social information processing in autism. Our findings challenge this interpretation: using non-social stimuli with high level of featural complexity (clocks with moving parts) as well as social stimuli with similar featural complexity allowed us to test whether there is anything unique to processing of social stimuli when they are compared with complex non-social stimuli. We found that autistic traits and symptoms are associated with atypical processing of complex information, not specifically social information. Our findings therefore suggest that this heterogeneity might be at least partly driven by stimulus complexity. Slower learning might be captured more fully in experimental paradigms that use more complex stimuli and thus differences in habituation findings in the literature might be partly explained by this. Further, studies in habituation in autism have sometimes found null effects and they usually do not take into consideration the presence of co-occurring difficulties and conditions. In our study, autistic children (with and without autism) showed slower rates of change in look durations to both repeating and changing stimuli, irrespective of the type of stimulus. However, only autistic participants without ADHD showed prolonged attention to repetition reflecting a bias against novelty in contexts of higher stimulus complexity. Participants with autism with comorbid ADHD did not show this profile. This again implies that heterogeneous findings in the habituation literature in autism might be partly driven by lack of proper characterization of the co-occurring conditions in autistic participants. In our study, presence of ADHD appears to benefit autistic individuals by combating the biases against novelty that emerge when processing more complex stimuli.

Previous research has also shown that autistic children demonstrate an attentional preference towards revisiting previously explored regions at the cost of exploring new information (Pellicano et al., 2011; Elison et al., 2012; Gliga et al., 2018). These studies have used paradigms very different to ours, with multiple static objects present on the screen at once, both social and non-social. While our study does not refute those findings, we do question whether presence of information foraging biases of exploitation over exploration characterize autistic individuals in all contexts. In future studies, it would be important to manipulate stimulus complexity to assess whether the attentional biases reported in autism might be partly driven by slower processing of stimuli.

Given the cross-sectional nature of our study and the age groups we focused on (children and adolescents), we are limited in being able to shed light on specific mechanisms behind the differences observed in processing more complex stimuli and whether such differences are a consequence or a cause of autism. It has been suggested that habituation differences in autism might lead to an exaggerated perception of change, and that restricted and repetitive behaviors might be a resultant coping mechanism (Dawson and Lewy, 1989; Vivanti et al., 2018). Contrary to this, we found that differences in attention to changing stimuli in the Non-Social Complex condition (in Study 1) were associated with more social interaction impairments in children but were not related with restrictive, repetitive behaviours on the SCQ. Other studies have also found evidence for reduced habituation to complex stimuli to be linked with higher severity of social impairments (Kleinhans et al., 2009; Webb et al., 2010). This suggests that these differences in processing more complex stimuli are related to skills involved in social interaction, rather than RRBs. Social interaction is dependent on processing complex and ever-changing information in real time. Thus, development of social interaction differences might well be rooted in early differences in being able to process complex information. Further, Vivanti et al. (2018) found a similar bias against attending to changing information in preschoolers with autism, therefore these differences in attention and information processing might emerge quite early.

Importantly, given that biases against novelty were found in relation with stimulus complexity regardless of the social-ness of the information, it appears that domain-general models of mechanisms in autism rather than domain-specific models, such as those that focus on social processing atypicalities as a core mechanism in autism, are likely to hold more value. For instance, there is evidence for atypical functioning of dorsal and ventral attentional networks that support orienting of attention to novel information in autistic individuals (Gomot et al. 2006; Keehn et al., 2010; Farrant & Uddin, 2016). Early differences in the ability to shift attention (Elsabbagh et al., 2013) alongside atypical regulation of arousal (Orekhova & Stroganova, 2014; Klusek et al., 2015) might contribute to the development of an attentional style that prefers repetition over novelty, particularly when information is dynamic and complex, such as in social situations. Further research, particularly using longitudinal designs from an early age, is crucial to identify the precise mechanisms that drive such differences in attention and information processing and how these link with development of autism-specific symptoms.

There were some differences between the findings from our two studies. In the clinical study, prolonged attention to repetition and biases against attending to novelty were present only in the Non-Social Complex condition. In comparison, in the second study, we found this effect in both the Non-Social Complex and Social Conditions. In comparison, Vivanti et al. (2018) found similar differences in a younger sample with stimuli from the Non-Social Simple condition (the only condition they used). Many factors could have led to these discrepant findings. Firstly, we did not match the stimuli between conditions. Like most developmental studies, this is a difficult task to accomplish while trying to retain the natural-ness of stimuli. Rather, we manipulated complexity and social-ness of stimuli. Secondly, the children in Study 2 (Age range- 4–12 years, Mean Age: 101.8 months) were younger than Study 1 (Age range- 7–15 years, Mean Age: 129.6 months); both of whom were older than Vivanti et al (27)’s sample (Mean Age calculated for Autistic and neurotypical participants from their study: 46.78 months). Thirdly, Study 1 included clinical participants, children diagnosed with autism, while Study 2 included children with varying levels of traits of autism. Any of these factors could have led to the differences in our findings. Further research using big samples at different developmental time-points and including participants on either side of the diagnostic boundary is required to understand these subtle differences.

There were some limitations of the current study. Sample sizes in both Study 1 and Study 2 were modest. Specifically, in Study 1, while we were able to recruit 50 autistic participants, only 18 of these could be characterized as Autism-only, while 32 participants met criteria for co-existing ADHD. This is in line with rates of co-occurrence of autism and ADHD and highlight that co-existing ADHD is the norm rather than the exception in autism (Leitner 2014). However, careful characterization of the sample in this manner (not often done in autism research) removes sources of noise and thus improves statistical power. In Study 1, we also included children from another clinical group (ADHD) and found the results to be specific to children with autism, which makes the finding more robust. The replication of the main effects in samples of children with clinically significant symptoms of autism and children with higher traits of autism further improves confidence in our findings. Regardless, our findings warrant replication in larger and more representative samples.

Importantly, we found that differences in attention to changing information were related to context and the type of information being presented, and thus might be partly influenced by IQ. Our sample in Study 1 was unbalanced with regard to IQ, with clinical participants showing lower IQ than neurotypical participants. However, while IQ was partly associated with the main clinical effect, it did not explain completely the relationship between SCQ scores and differences in looking to changing stimuli in the Non-Social Complex condition (the partial correlation did not reach statistical significance but the correlation was still present and indicated an effect size of similar magnitude). Further, the autistic participants with co-occurring ADHD had lower IQ than those without; yet the pattern of differences was specific to autistic children without co-occurring ADHD. In Study 2, we did not find any relationship between BPVS scores and looking to more complex repeating or changing stimuli. Therefore, while IQ might contribute to these differences in processing more complex stimuli, from our data it appears that IQ does not fully explain these differences. Other studies in the literature have also found information foraging biases such as in our study not to be associated with IQ (Pellicano et al., 2011; Elison et al., 2012). Therefore, information foraging biases might be independent of IQ in these populations. Another possible limitation of this study is the nature of stimuli used, particularly in the non-social complex condition. The clocks we used were not naturalistic and it is possible that given the prevalence of digital clocks these days, the effects we saw are driven partly by lack of familiarity with these stimuli. However, this is still important to further investigate since lack of familiarity might influence foraging differently in autistic individuals than non-autistic individuals. Importantly, clocks contain many small features each of which have symbolic meanings and they are typically processed by paying closer attention to these local features. On the other hand, faces are typically processed more globally (Gao et al., 2011). It is possible that the pattern of differences is related to this, given that there are differences in local versus global processing in autism (Koldewyn et al., 2013). However, if this were the case, those with autism would have shown better processing of the clocks instead of the other two conditions so we do not believe this to be the case. Future studies should use different types of complex non-social and social stimuli to investigate these effects further, using designs which balance social-ness and complexity for both social and non-social stimuli (for example, stimuli of varying levels of complexity in other modalities such as the auditory modality, static and dynamic social and non-social stimuli, unimodal and multimodal social and non-social stimuli, etc.).

In conclusion, our research demonstrated that reduced attention to changing information might emerge only in conditions with higher stimulus complexity in autistic individuals and in typically developing children with high autistic traits (regardless of the stimuli being social or non-social). This is an important finding and future research should look at when such differences first emerge and how they develop over time in interaction with symptoms of autism.

## Supplementary Information

Below is the link to the electronic supplementary material.

Supplementary file1 (DOCX 26 kb)

## Data Availability

The datasets analysed in the current study as well as example videos of stimuli are available currently at https://osf.io/v9fex/. Raw eye-tracking files and videos used in the task are available from the corresponding author upon reasonable request.
