# Supplementary material for: What is the Effect of Stimulus Complexity on Attention to Repeating and Changing Information in Autism?
Source: J Autism Dev Disord. 2021 Mar 19;52(2):600–16. doi: 10.1007/s10803-021-04961-6 (PMC8813872; doi:10.1007/s10803-021-04961-6)
Supplement: Supplementary file 1 — Supplementary file1 (DOCX 26 kb) [file 10803_2021_4961_MOESM1_ESM.docx]

**Supplementary Information**

Task Information

We created two versions of the task: in one version of the task, the repeating stimulus in the social condition was male and in the other, it was female, in case stimuli of different genders elicited different attentional effects depending on the gender of the participant. For the non-social conditions (Simple and Complex), each version of the task used a different stimulus as the repeating stimulus. Each participant did one version of the task, and we presented the version with the male repeating social stimulus to half the participants and the version with the female social repeating stimulus to the other half. Analyses on the main dependent task variables confirmed no significant differences between task versions and so we collapsed across the versions in all analyses.

**Study 1**

Sample Characteristics and Study Procedure

Participants were included in the Autism group if they presented with clinically significant symptoms of autism on the ADOS-2 (ADOS comparison scores > 4), the DAWBA (meeting DSM-5 and ICD-10 criteria) (American Psychiatric Association 2013; World Health Organization 1993) and SCQ (raw score > 15) and a consensus clinical review of all available information applied to ensure diagnostic rigor (McEwen et al. 2016).

Participants were included in the ADHD group if they presented with clinically significant symptoms of ADHD combined presentation on DAWBA (meeting DSM-5 criteria) (American Psychiatric Association 2013) and the CRS (T scores > 65) and a consensus clinical review of all available information. Importantly, where we did not have teacher CRS on a child and the child did not have a pre-existing diagnosis of ADHD, they were not included in the study since presence of these symptoms across different settings is important for a diagnosis.

Participants were included in the comorbid Autism + ADHD group if they met research diagnostic criteria for both autism and ADHD as defined above.

Participants were excluded from the neurotypical group if any of these measures revealed clinically significant symptoms (as defined above), or significantly elevated risk (i.e., >75% probability) of presence of any DSM-5 or ICD-10 diagnoses as predicted by DAWBA, or there was family history of ADHD or autism. Children with ADHD were excluded if they were on non-stimulant medications or if their parents did not wish to remove them from stimulant medications for 24 hours before the study.

Other exclusion criteria were neurological disorders including epilepsy and Tourette’s syndrome and non-fluent English in the child or parent. Other mental health conditions (anxiety, depression, obsessive-compulsive disorder, conduct disorder, oppositional defiant disorder etc.) and intellectual disability were not excluded. Another aim of this research study, not covered within this paper, was to investigate the role of IQ (intelligence quotient, as measured by WASI) in attention in Autism and ADHD. Therefore, participants were not excluded for having intellectual disability. None of the participants included in the present paper had IQ below 70, 3 participants had IQ below 80.

After providing informed consent, parents completed DAWBA, SCQ and CRS-3 as well as demographic and medical information. Participants with ADHD who were taking stimulants were asked to withdraw from medication for at least 24 hours prior to the laboratory session. Participants completed the ADOS and WASI-II and those who met the inclusion criteria then completed the eye-tracking and EEG batteries. At the end of the study, participants were given a certificate and a £15 voucher. Parents’ travel expenses were reimbursed.

Number of fixations (control variable measuring task engagement)

Follow-up pairwise comparisons were conducted to evaluate the interaction of Condition*Autism, to identify whether within Condition (Non-Social Simple, Non-Social Complex, Social), there were differences between groups with and without Autism in number of fixations to the screen. At each level of Condition, there were no significant differences between groups on this variable:

Non-Social Simple Condition: Groups with Autism (Mean ± S.E. = 79.09 ± 2.71) demonstrated similar number of fixations to the screen as those without Autism (Mean ± S.E. = 81.52 ± 2.55); p= .52.

Non-Social Complex Condition: Groups with Autism (Mean ± S.E. = 73.63 ± 3.11) demonstrated similar number of fixations to the screen as those without Autism (Mean ± S.E. = 76.64 ± 2.92); p= .48.

Social Condition: Groups with Autism (Mean ± S.E. = 88.95 ± 2.86) demonstrated similar number of fixations to the screen as those without Autism (Mean ± S.E. = 82.66 ± 2.69); p= .11.

**Study 2**

Results on the main dependent variable- Rate of change in look durations (after excluding children with Autism or ADHD from the sample)

There was a main effect of Stimulus (F (1, 58) = 7.41, p = .009, ƞ^2^_p_ = .113); with the slope to the repeating stimuli being more negative (Mean ± S.E.= -.89 ± 6.59) than the slope to the changing stimuli (Mean ± S.E.= 54.13 ± 7.7). This was modulated by a Condition*Stimulus interaction (Greenhouse-Geisser F (1.78, 103.336) = 5.389, p = .008, ƞ^2^_p_ = .085). The main effect of Stimulus was present within each condition: Simple (Mean difference (Repeating vs Changing) = -66.206 ± 23.87, p = .007); Complex (Mean difference = -67.34 ± 29.81, p < .028); Social (Mean difference = -58.73 ± 14.296, p < .001) (See Fig. 5a). This interaction was further moderated by a 3-way interaction with AQ (F (1.78, 103.336) = 5.945, p = .005, ƞ^2^_p_ = .093). As can be seen below in Figure 5b, in both the Non-Social Complex and Social conditions, the main effect of Stimulus reversed, such that in the Non-Social Complex and Social conditions, those with higher AQ scores (i.e., higher levels of autistic traits) showed longer look durations to the repeating stimuli over time and reduced look durations to the changing stimuli over time.

Figure 5a. Interaction between Condition and Stimulus on rate of change in look durations

Figure 5a Legend: Bars show the mean (±1 standard error) coefficient of the slope for the rate of change in look durations over trials (plotted on the y-axis). These data are split by stimulus type and condition. Asterisks denote statistical significance: *p<.05, **p<.01, ***p<.001

Figure 5b. Interaction between Condition, Stimulus and AQ on rate of change in look durations

Figure 5b Legend: Bars show the mean (±1 standard error) coefficient of the linear relationship between scores on the Autism Spectrum Quotient- Child Version (AQ-Child) and the rate of change in look durations over trials (plotted on the y-axis). These data are split by stimulus type and condition.

Bias-corrected and accelerated bootstrapped correlations of BPVS and Age with AQ and Rate of change in look durations to repeating and changing stimuli in Non-SocialComplex and Social Conditions

|  | AQ-Child | Rate of Change in Look Durations over Trials | | | |
| --- | --- | --- | --- | --- | --- |
|  |  | Non-Social Complex Repeating Stimulus | Non-Social Complex Changing Stimulus | Social Repeating Stimulus | Social Changing Stimulus |
| BPVS standard score | r = -.02, p = .88, [-.28, .25] | r = -.08, p = .55, [-.37, .24] | r = .16, p = .21, [-.08, .38] | r = -.02, p = .87, [-.2, .15] | r = -.02, p = .89, [-.26, .23] |
| Age (in months) | r = -.12, p = .35, [-.39, .19] | r = -.09, p = .51, [-.31, .2] | r = .01, p = .94, [-.25, .26] | r = .08, p = .53, [-.18, .34] | r = -.1, p = .44, [-.36, .18] |

BPVS: British Picture Vocabulary Scale, Third Edition, Standardized scores; AQ-Child: Autism-Spectrum Quotient- Child’s Version; []= Bootstrapped and bias-corrected 95% confidence intervals around the Pearson’s correlation r.

References

American Psychiatric Association (2013). *Diagnostic and statistical manual of mental disorders (DSM-5®)*: American Psychiatric Pub.

McEwen, F. S., Stewart, C. S., Colvert, E., Woodhouse, E., Curran, S., Gillan, N., et al. (2016). Diagnosing autism spectrum disorder in community settings using the Development and Well-Being Assessment: validation in a UK population-based twin sample. *J Child Psychol Psychiatry, 57*(2), 161-170, doi:10.1111/jcpp.12447.

World Health Organization (1993). *The ICD-10 classification of mental and behavioural disorders: diagnostic criteria for research* (Vol. 2): World Health Organization.
